# A pilot study about on-farm assessment of health and welfare in rabbits kept in different housing systems

**DOI:** 10.3389/fvets.2022.936643

**Published:** 2022-08-11

**Authors:** Angela Trocino, Francesca Menegon, Cristina Zomeño, Dario Pasqualin, Giovanni Cunial, Gerolamo Xiccato, Fabrizio Pirrone, Daniela Bertotto, Martina Bortoletti, Francesco Dorigo, Antonio Lavazza, Guido Di Martino

**Affiliations:** ^1^Department of Comparative Biomedicine and Food Science (BCA), University of Padova, Padova, Italy; ^2^Department of Agronomy, Food, Natural Resources, Animal and Environment (DAFNAE), University of Padova, Padova, Italy; ^3^Istituto Zooprofilattico Sperimentale Delle Venezie, Padova, Italy; ^4^Institute of Agrifood Research and Technology (IRTA)-Food Quality and Technology Program, Monells, Spain; ^5^Rabbit Sector Veterinary Practice, Bassano del Grappa, Italy; ^6^Istituto Zooprofilattico Sperimentale della Lombardia e dell'Emilia Romagna, Brescia, Italy

**Keywords:** animal-based indicators, performance, stress, behavior, cages, parks

## Abstract

This pilot study tested an on-farm protocol based on resource, management, and animal-based measures to evaluate the on-farm health and welfare of rabbits kept in four different housing systems. In detail, the four housing systems were (1) standard breeding cages for reproducing does (3,300 cm^2^) with their litters associated with bicellular cages for growing rabbits (1,200 cm^2^); (2) dual-purpose cages for both reproducing does and growing rabbits (3,655 cm^2^); (3) enriched cages (4,739 cm^2^) for both reproducing does and growing rabbits equipped with a wire-mesh elevated platform (1,015 cm^2^); (4) parks (30,977 cm^2^) made up of four modules (7,744 cm^2^ each) joined by removing the wire net walls between them with growing rabbits kept in collective parks and reproducing does individually in the single modules. A total of 12 commercial farms (three farms/four housing systems) were visited during three seasons (summer, autumn, and winter) on two occasions each: (1) a pre-weaning visit for recordings on reproducing does and litters and (2) a pre-slaughtering visit for recordings on growing rabbits. At the pre-weaning visit, the prevalence of health concerns did not differ among does and litters kept in the different housing systems. At the pre-slaughtering visit, a higher prevalence of dermatomycosis was found in farms with dual-purpose cages and parks. Overall, taking into account the limitations due to the small sample size per housing system and the field conditions, the on-farm assessment tested in the present pilot study did not highlight major differences in the welfare and health of reproducing does and their kits as well as of growing rabbits in farms using different housing systems, which need to be confirmed on a large number of farms. The study also outlined the role of several management and environmental factors changing from one farm to another, which stresses the troubles of accounting for on-farm rabbit welfare and health exclusively to the housing system.

## Introduction

The Farm to Fork strategy (1) of the European Green Deal (2) calls for new and revised regulations for the protection of the welfare and health of farmed animals. As for rabbits, during the last decades, consumers' concerns about farming practices and housing systems have grown (3, 4). The European Parliament Resolution (5) on minimum standards for the protection of farmed rabbits called for alternatives, which were definitively stated by the European Parliament Resolution on the European Citizens' Initiative End the Cage Age (6), asking the Commission to phase out cages in all European farms, possibly by 2027, for any farmed animals.

In Europe, commercial farms of meat rabbits are mostly located in Spain, France, and Italy, which account for 83% of the European production (7, 8), while in many other countries, rabbits are popular only as pets. Farming of meat rabbits shows a wide variability both among and within countries (9, 10). The majority of commercial farms use cages, i.e., standard breeding cages for reproducing does with their litters associated with bicellular cages for growing rabbits, or dual-purpose cages for both reproducing does with their litters and growing rabbits. Some farms use structurally enriched cages (11), whereas a few commercial farms use alternative systems based on parks (also called elevated pens) that can house growing rabbits in large groups (about 30–35) and reproduce does with their litters individually or, seldom, in continuous or part-time groups (11, 12). Park housing systems have been tested during the last decades but are not yet widespread or validated at a commercial level all over Europe, for which the technical standards for their implementation are not yet fully available (5). Park systems have also shown some weaknesses in terms of health and welfare of growing rabbits due to aggression and diseases transmitted through the oro-fecal route (5, 7), besides being associated with elevated levels of aggression and stress when reproducing does are reared in groups (11, 12). From the perspective of a cage phasing-out, these issues generate deep uncertainty in farmers and technicians, as the rabbit sector has also been hit hard with the decline in meat consumption and the economic crisis. Sales prices have fallen by ~20% in 3 years, while production costs are significantly and continuously increasing (5). There is only one study published about the economic performances of rabbit farms. It shows that enriched cages are economically sustainable and comparable to conventional housing systems with bicellular or dual-purpose cages and provide a significant reduction in drug use in the tested farms (13). At the same time, no information is available about farmer perception and willingness to change which could be driving factors for adapting production systems to rabbit welfare needs.

The latest Scientific Opinion of the European Food and Safety Authority (EFSA) (11) compared the health and welfare of rabbits kept in different housing systems by a global impact score, based on both health- and behavior-related welfare consequences, obtained through an expert knowledge elicitation (EKE) process. The probabilistic analyses of EKE results showed that cage systems are likely associated with lower rabbit welfare, mainly because of behavioral restrictions and concerns. However, field data about the prevalence of welfare consequences are missing. Moreover, unlike other species, no validated animal-based measures (ABMs) or protocols to assess on-farm animal welfare are available for rabbits yet (14). Some measures and protocols have recently been tested only on farms using standard barren cages (15, 16). Therefore, this pilot study aimed to provide a preliminary evaluation of on-farm health and welfare of rabbits kept in different housing systems based on a protocol using resource-, management-, and animal-based measures, along with including a number of the few commercial farms that were using alternative systems. In detail, the protocol was tested in the following housing systems: (1) the standard cage system, using standard breeding cages for reproducing does with their litters associated with bicellular cages for growing rabbits; (2) the dual-purpose cage system, where dual-purpose cages are used for both reproducing does with their litters and growing rabbits; (3) the enriched cage system, based on enriched cages for both reproducing does with their litters and growing rabbits; and (4) the park system, which uses single modules of a park for reproducing does with their litters and four joined modules as a larger park for growing rabbits.

## Materials and methods

### Farms and housing systems

A total of 12 commercial farms located in the Northeast of Italy took part in the assessments. All farms were closed-cycle, with a population size between 456 and 3,890 reproducing does.

These farms were proposed by practitioners working in the field, based on the farmers' availability, to have a sample of three farms per housing system, i.e., (1) the standard cage system; (2) the dual-purpose cage system; (3) the enriched cage system; and (4) the park system.

In the case of the standard cage system, at weaning, the litters were moved from the breeding cages to the bicellular cages, while the reproducing does always remained in the original cages. In these farms, the size of the standard breeding cage was 3,300 cm^2^, whereas the size of the bicellular cage was 1,200 cm^2^ (Table 1).

**Table 1:** Housing systems and cage size in the farms subjected to the on-farm welfare evaluation in reproducing does with their litters and in growing rabbits.

**Housing system**	**Standard-cage system**		**Dual-purpose–cage system**	**Enriched-cage system**	**Park system**	
**Type of cage**	**Breeding cage**	**Bicellular cage**			**Single module**	**Park (four modules)**
**Rabbit category**	**Reproducing does with their litters**	**Growing rabbits**	**Growing rabbits; reproducing does with their litters**	**Growing rabbits; reproducing does with their litters**	**Reproducing does with their litters**	**Growing rabbits**
Total available surface (cm^2^)^*^	3,300	1,200 (1008–1,584)	3,655 (3,315–3,927)	4,739 (4,522–5,082)	-	30,977 (30,814–31,304)
Available surface/rabbit (cm^2^)	3,300	600	609	592	-	860
Growing rabbits (n/cage)	-	2	6	8	-	36 (32–40)
Growing rabbits (n/m^2^)	-	17 (13–20)	16 (15–18)	17 (16–18)	-	12 (10–13)
Live weight at slaughtering (kg/m^2^)	-	46.0 (33–56)	44.0 (40–49)	44.1 (39–47)	-	30.1 (29–32)

* Including the nest area and the platform surface when available.

In the farms with the other housing systems, dual-purpose cages were smaller (3,655 cm^2^) than enriched cages (4,739 cm^2^). Enriched cages were equipped with a wire-mesh elevated platform (1,015 cm^2^). Parks (30,977 cm^2^) were made up of four modules (each 7,744 cm^2^) joined by removing the wire net walls between them (Table 1). Parks had a plastic-mesh platform (2,282 cm^2^ for a single module and 9,129 cm^2^ for a park) and a plastic-slatted floor (Supplementary Figure 1).

In farms using the dual-purpose cage, enriched cage, and park systems, at weaning, the does were moved to clean cages or to clean individual modules of the parks, while the litters remained where they were born until slaughtering. In the farms using parks, at weaning, four contiguous modules were joined by removing wire walls between them to obtain parks in which growing rabbits from four/five litters were kept until slaughtering in large groups (32–40 rabbits).

As detailed in Supplementary Table 1, besides the housing system, farms differed in several other factors, such as animal genotype (Hyla, Grimaud, or Martini commercial crossbreed), reproduction rhythm (does artificially inseminated at 11 days or 18 days after kindling), building type (indoor or semi-plein air), ventilation system (extraction with/without cooling system), and the presence of plastic mats in the cages, diets, and feeding programs for growing rabbits (*ad libitum* or restricted). The weaning age of litters ranged from 32 to 38 days and the slaughtering age of growing rabbits ranged from 71 to 86 days, due to market requirements, besides the farm's own organization. Within the different housing systems, it should be noted that (1) only one farm using enriched cages adopted the genotype Martini; (2) farms using enriched cages adopted only the reproduction rhythm with insemination 11 days after kindling; (3) farms with standard cages did not use foot mats in cages for reproducing does; (4) farms with enriched cages and parks only used *ad libitum* feeding for growing rabbits, while, in farms with standard and dual-purpose cages, both feeding systems were used. These issues have been taken into account in the discussion of results.

### On-farm recordings and sampling

Recordings were scheduled to cover three seasons (i.e., autumn, winter, and summer) with two visits per season per farm: (1) a pre-weaning visit, the week before weaning (27–31 days after kindling) for recordings on reproducing does and litters, and (2) a pre-slaughtering visit, 2–5 days before slaughtering for recordings on the corresponding growing rabbits.

Resource and management-based indicators besides ABMs were recorded in does with their litters on the pre-weaning visit (Table 2) and in growing rabbits on the pre-slaughtering visit (Table 3). On each visit, farm temperature and humidity were measured using an Anemometer Kestrel 5000 (Nielsen-Kellerman Company, Boothwyn, PA, USA); NH_3_ e CO_2_ gases were recorded by a Gas Detector X-am 7000 (Draeger, Lübeck, Germany).

**Table 2:** Health and behavioral animal-based indicators and resource- and management-based data recorded on farms with reproducing does and kits the week before weaning (pre-weaning visit).

**Sample size**	**Indicator type**	**Indicators**	**Scores**
75 reproducing does with their litters /farm/visit	Resource-based	Cage or park system	Standard breeding cage, dual purpose cage, enriched cage, a single module of parks
		Cage characteristics	Footrest presence (yes/no)
		Temperature, relative humidity, NH_3_, and CO_2_ concentrations	Measurements at 5 locations in the barn (4 lateral and 1 central)
	Management-based	Animal genotype	-
		Reproductive rhythm	11 d after kindling /18 d after kindling
		Weaning age	-
	Animal-based	Doe physiological status	Primiparous/pluriparous
		Doe body weight	-
		Doe body condition score (BCS)	Five-point scale (0–4; 0: cachexia; 4: obesity) (17)
		Doe health concerns:	
		Respiratory symptoms	Nasal and/or ocular secretion (yes/no)
		Diarrhea	Yes/no
		Mastitis	
		Ulcerative pododermatitis	yes/no and severity (1: minor and limited lesions; 2: extended lesions; and 3: deeper, extended, and open lesions)
		Dermatomycosis	
		Litter weight	
		Litter size	
		Kit health concerns	
		Respiratory symptoms	Nasal and/or ocular secretion (yes/no)
		Diarrhea	yes/no
		Dermatomycosis	yes/no
		Kit mortality	Average data of the entire productive cycle (provided by the farmer)

**Table 3:** Health and behavioral animal-based indicators and resource- and management-based data recorded on farms with growing rabbits before slaughtering (pre-slaughtering visit).

**Sample size**	**Indicator type**	**Indicators**	**Scores**
100 growing rabbits/farm/visit	Resource-based	Cage or park system	Standard bicellular cage, dual purpose cage, enriched cage, parks
		Cage characteristics	Available surface (cm^2^)
		Stocking density	Animals/cage, animals/m^2^, kg/m^2^
		Temperature, relative humidity, NH_3_, and CO_2_ concentrations	Measurements at 5 locations in the barn (4 lateral and 1 central)
	Management-based	Animal genotype	
		Feeding system	*Ad libitum* / restricted
		Slaughtering age	
	Animal-based	Body weight	
		Health concerns:	
		Respiratory symptoms	Nasal and/or ocular secretion (yes/no)
		Diarrhea	Yes/no
		Injuries associated to aggression	Yes/no and severity
		Mortality	Average data of the entire productive cycle (provided by the farmer)

At every pre-weaning visit, for a random sample of 75 does (12–15 at their first kindling), the does' body weight, body condition score (BCS), and health status were individually evaluated (Table 2). The BCS was assessed by palpating the fullness of muscle and fat in the lumbar and gluteal regions based on a five-point scale (0–5) (17). Symptoms related to respiratory (nasal and/or ocular secretion) and digestive (diarrhea) problems, mastitis, ulcerative pododermatitis, and dermatomycosis were also scored. The litter size and weight and the kit health (symptoms of respiratory and digestive problems, dermatomycosis) were also assessed (Table 2).

During the pre-slaughtering visits, body weight, signs of diarrhea, and lesions related to aggression and dermatomycosis were individually assessed on a random sample of 100 growing rabbits per visit (2 rabbits each × 50 bicellular cages, dual-purpose cage, and enriched cages; 20 rabbits × 5 parks) (Table 3).

By the end of the trial, out of the initially selected 12 farms, one farm with a dual-purpose cage system was available only for two seasons (i.e., two pre-weaning and two pre-slaughtering visits in autumn and winter) and one farm with parks was available only for one season (i.e., one pre-weaning visit and one pre-slaughtering visit in autumn). Health data of growing rabbits were not recorded in autumn because of the unavailability of some farmers.

Recordings ran from September 2018 to August 2019. The pre-weaning visits lasted on average 90 min, while the pre-slaughtering visits took 60 min. Both types of visits involved two assessors.

In the autumn and summer seasons, while weighing, hair samples were collected from 10 animals at random per visit from both reproducing does at the pre-weaning visits and from growing rabbits at the pre-slaughtering visits. Hair was gently collected using a brush from rabbits' back region and hind legs, individually packed in plastic bags, and soon transferred to the labs of the University of Padova, where they were stored at −20°C until analysis for cortisol.

### Hair cortisol analysis

Hair samples (50 mg) were homogenized in a mortar with pestle and liquid nitrogen, mixed with 5 ml of absolute methanol, and placed at 50°C in an oven for 18 h. Next, the tubes were centrifuged for 15 min and the supernatant was brought to dryness in a nitrogen stream. The dry extract was recovered with phosphate buffer and loaded onto a microplate for the cortisol assay. The antibody anti-cortisol used (Analytical Antibodies, Bologna, Italy) had the following cross-reactivities: cortisol 100%, prednisolone 44.3%, 11-deoxycortisol 13.9%, cortisone 4.95%, corticosterone 3.5%, prednisone 2.7%, 17-hydroxyprogesterone 1.0%, 11-deoxycorticosterone 0.3%, dexamethasone 0.1%, progesterone <0.01%, 17-hydroxypregnenolone <0.01%, and pregnenolone <0.01). At the validation tests, the regression curve between the steroid concentration and the reciprocal of the dilution factor showed good parallelism (y = 19.3x - 0.2; R^2^ = 0.999); optimal results were also obtained for repeatability (intra-assay CV = 3.6%) and extraction yield (76%).

### Statistical analysis

All data were analyzed using SAS 9.4 software (SAS, 2013). Performance data of does, litters, and growing rabbits were given as input to an ANOVA using the MIXED procedure and by fitting the linear mixed model with housing system (standard cage; dual-purpose cage; enriched cage; park), season (autumn, winter, and summer), and their interaction as fixed effects and the farm as a random effect to account for the specificity of each farm with all the different production factors within a farm. The structure variance components were used to model variance and covariance matrices.

Data related to the prevalence of health concerns were first coded as binary variables (YES/NO). Then, the average prevalence per farm and per cycle was calculated and data were given as the percentage of animals affected by a health concern with respect to the total number of animals assessed per visit per farm. Prevalence data were analyzed with the GLIMMIX procedure of SAS with a model considering housing system, season, and their interaction as the main effects. A Poisson distribution was assumed for these data.

Then, to explore the possible effects of the different production factors besides the housing system, a risk factor analysis (18, 19) for performance data was carried out using the GLM procedure of SAS and by fitting a model with housing system, season, animal genotype, reproductive rhythm, parity order, and footrest presence for reproducing does and feeding system (restriction or not) for growing rabbits. For health prevalence data, the same model was fitted with the GLIMMIX procedure, assuming a Poisson distribution for these data.

Lastly, hair cortisol contents of reproducing does and growing rabbits were analyzed using the MIXED procedure and by fitting a model with housing system, season (autumn and summer), and their interaction as fixed effects and the farm as a random effect. The structure variance components were used to model variance and covariance matrices.

## Results

### Pre-weaning visit

At the first visit, average temperatures were rather similar among farms using different housing systems (Table 4). The lowest minimum value (12.5°C) was recorded in farms with the standard cage system, whereas the maximum temperature ranged from 24.7°C in farms with the park system to 28.5°C in farms with the standard cage and enriched cage systems. The average relative humidity values were similar among farms (64.0–67.6%) (Table 4). The highest levels of CO_2_ and, especially, ammonia were recorded in farms with the standard cage and dual-purpose cage housing systems. Ranges of variations from minimum to maximum values for air gases were quite large within and among housing systems.

**Table 4:** Results of the pre-weaning visit in farms with different housing systems across three seasons: environmental (means and intervals) and animal-based measures (means) in reproducing does and kits.

	**Housing system**					**Season**				**RMSE**
	**Standard cage**	**Dual-purpose cage**	**Enriched cages**	**Parks**	***P-*value**	**Autumn**	**Winter**	**Summer**	***P*-value**	
**Environmental data**										
Visits (no)	9	8	9	7		12	11	10		
Temperature (°C)	21.3 (12.5–28.6)	20.1 (14.3–26.6)	21.7 (17.0–28.5)	20.1 (14.4–24.7)	-	21.1 (18.9–24.9)	15.7 (12.5–18.4)	26.5 (24.6–28.6)	-	-
Relative humidity (%)	67.6 (55.7–79.4)	65.5 (54.0–76.7)	63.9 (35.2–79.4)	64.0 (55.1–77.6)	-	66.4 (55.7–79.4)	58.4 (35.2–71.8)	72.2 (55.1–79.4)	-	-
CO_2_ (ppm)	1,042 (500–1,914)	1,260 (480–1,880)	986 (100–1,740)	1,000 (540–1,420)	-	1,103 (100–1,914)	1,707 (1,420–1,880)	656 (480–1280)	-	-
NH_3_ (ppm)	9.9 (0.0–31.4)	10.7 (2.8–21.2)	4.6 (1.0–7.2)	6.3 (2.0–9.6)	-	9.0 (0.0–31.4)	12.9 (6.6–17.6)	3.9 (1.8–7.2)	-	-
Kit mortality (%)	5.3 (0.0–14.0)	5.7 (3.0–9.0)	5.0 (0.0–15.0)	5.0 (3.0–8.0)	-	3.0 (0.0–5.0)	7.6 (3.0–15.0)	5.3 (2.0–14.0)	-	-
**Animal-based measures**									
Does with litters (no.)	675	600	675	300		825	750	675		
Days after kindling	29.4	28.0	28.6	28.5		28.7	28.9	28.9		
Doe weight (g)	4431^a^	4765^b^	4914^c^	4968^c^	<0.001	4841^c^	4775^b^	4566^a^	<0.001	479.7
Doe BCS	1.91^a^	1.94^ab^	2.00^b^	2.09^c^	<0.001	1.92^a^	1.98^b^	2.01^b^	0.006	0.496
Litter size (no.)	8.08^a^	8.21^a^	8.61^b^	9.18^b^	<0.001	8.24^a^	8.83^b^	8.19^a^	<0.001	1.044
Kit weight (g)	614	626	610	614	0.10	616^b^	641^c^	588^a^	<0.001	106.1
**Doe health concerns (%)** ^*^										
Diarrhea	7.0	5.2	5.4	6.3	0.096	7.7^a^	3.5^b^	6.7^a^	0.003	-
Pododermatitis	2.8	7.9	1.0	0.0	1.000	4.4	4.5	0.3	0.999	-
Mastitis	2.8	6.1	1.0	0.0	0.080	3.0	1.6	3.7	0.999	-
Dermatomycosis	2.8	3.2	2.8	2.7	1.000	0.0	0.4	9.2	0.999	-
Respiratory symptoms	0.6	0.2	0.1	0.0	1.000	0.3	0.3	0.30	1.000	-
Litter health concerns (%)*										
Diarrhea	0.7	0.7	0.7	3.0	1.000	2.4	0.1	0.3	0.999	-
Dermatomycosis	2.4	3.8	0.0	0.0	1.000	0.7	3.9	0.6	1.000	-

RMSE, root mean square error of the model; BCS, body condition score (0: cachexia, 4: obesity).

*Percentage of animals affected with respect to the total assessed on each visit per farm.

^a, b, c^Means with different letters on the same row significantly differ across the housing system or season (P-value < 0.05, Bonferroni test).

As for ABMs (Table 4), the reproducing does in the farms with the standard cages showed the lowest live weight (4,431 vs. 4,765 g vs. 4,914 and 4,968 g; *P* < 0.001) compared to the does in the farms using the dual-purpose cage and, especially, enriched cage and park systems, while BCS was the lowest in does kept in farms with standard cages compared to those kept in farms with enriched cages and parks (1.91, 1.94, 2.00, and 2.09; *P* < 0.001). As for litter size, the lowest values were found in the farms using the standard cage and dual-purpose cage systems compared to those using the enriched cage and park systems (8.08 and 8.21 vs. 8.61 and 9.18; *P* < 0.001). The prevalence of health concerns did not differ among does or their litters kept on farms with different housing systems (Table 4). The average prevalence of diarrhea in the does ranged from 5.2 to 7.0%, pododermatitis lesions ranged from 0.0 to 7.9%, mastitis ranged from 0.0 to 6.1%, dermatomycosis ranged from 2.7 to 3.2%, and respiratory symptoms ranged from 0.0 to 0.6% without significant differences among housing systems (Table 4).

As for the effect of season, the does were heavier in autumn and lighter in summer (4,841 vs. 45,66 g) with intermediate values in winter (4,775 g; *P* < 0.001). The kits were lighter in summer as well and heavier in winter than in autumn (588 vs. 616 vs. 641 g *P* < 0.001). In contrast, no influence of the season on health issues was observed, except for diarrhea in does, which had a higher prevalence in autumn and summer than in winter (7.7% and 6.7 vs. 3.5%; *P* < 0.01) (Table 4).

The analysis of the risk factors for the performance of reproducing does and kits confirmed the significant effects of the housing system and season, besides genotype, reproductive rhythm, doe parity order, and footrest presence (Table 5; Supplementary Table 2). Parity order was a risk factor for pododermatitis and dermatomycoses, whereas footrest presence played a role in pododermatitis occurrence.

**Table 5:** Risk factors (*P-values*) for animal-based measures in reproducing does and kits at the pre-weaning visit in farms with different housing systems across three seasons.

**Variation factors**	**Housing system**	**Season**	**Animal genotype**	**Reproductive rhythm**	**Parity order**	**Footrest presence**
	**Standard/ Dual-purpose/ enriched/park**	**Autumn/ Winter/ Summer**	**Grimaud/ Hyla/ Martini**	**11 d after kindling/ 18 d after kindling**	**Primiparous/ pluriparous**	**Yes/no**
**Doe**						
Live weight	<0.001	<0.001	<0.001	0.37	<0.001	<0.001
Body condition score	<0.001	0.003	0.085	0.013	0.212	<0.001
Diarrhea	0.066	0.006	0.146	0.207	0.833	0.556
Pododermatitis	0.710	0.001	0.869	0.001	0.029	0.005
Mastitis	0.521	0.042	0.301	0.025	0.55	0.177
Dermatomycosis	0.003	<0.001	0.044	0.013	0.002	0.021
**Litter**						
Litter size	<0.001	<0.001	<0.001	<0.001	<0.001	<0.001
Kit weight	<0.001	<0.001	<0.001	0.071	0.233	0.465
Diarrhea	0.679	0.005	0.591	0.509	0.604	0.259
Dermatomycosis	0.999	<0.001	0.122	0.002	0.989	0.991

**Figure 1:**
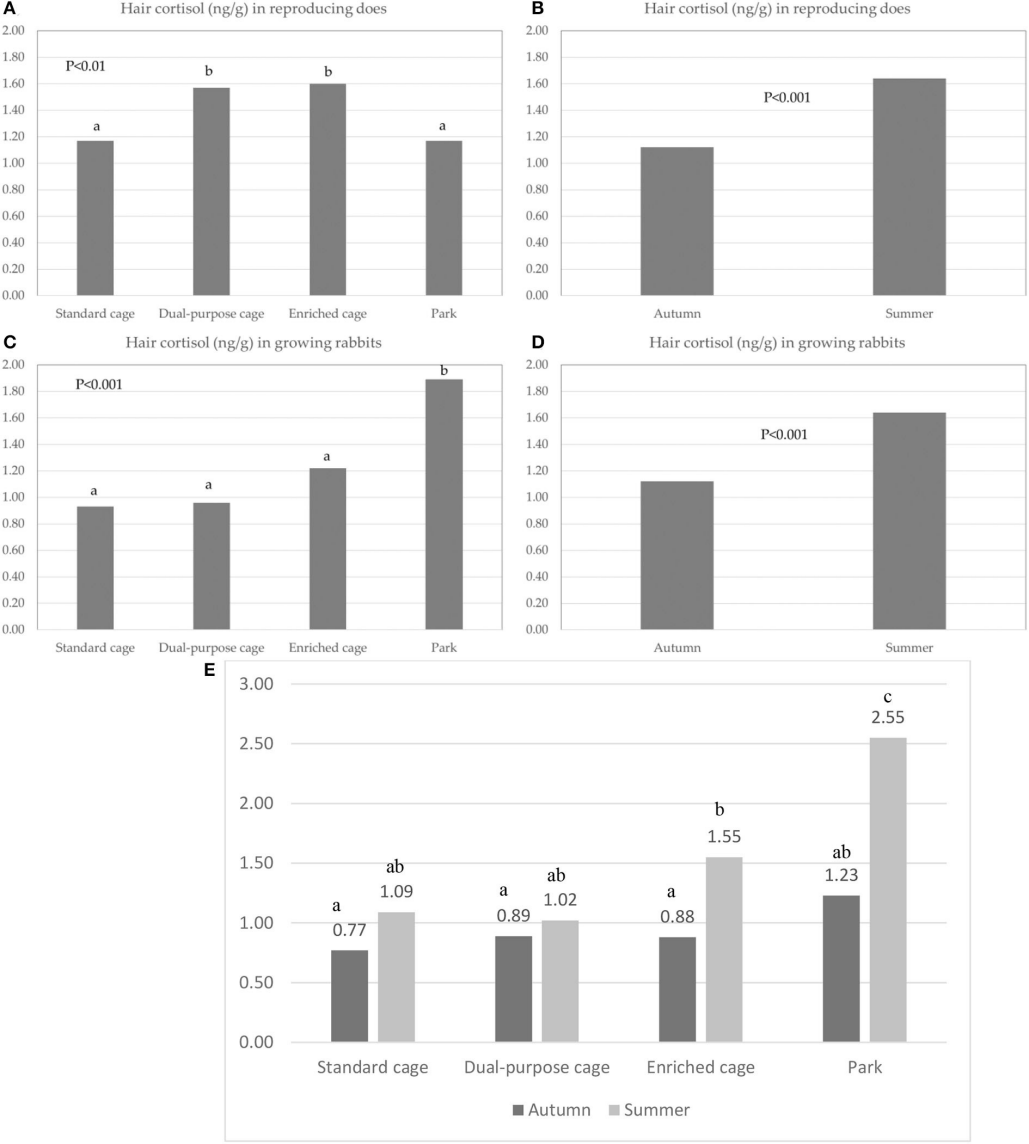
Hair cortisol content (ng/g): effect of the housing system **(A)** and the sampling season **(B)** in reproducing does; effect of the housing system **(C)**, the sampling season **(D)**, and the interaction between housing systems and sampling season **(E)** in growing rabbits. Different letters indicate a significant difference at *P*-value < 0.05.

Hair cortisol level in reproducing does was lower in the farms using the standard cage and park systems (1.17 ng/g) than in those using the dual-purpose cage and enriched cage housing systems (1.57 and 1.60 ng/g; *P* < 0.01) (Figure 1A) and on samples collected in autumn compared to those collected in summer (1.12 vs. 1.64 ng/g *P* < 0.001) (Figure 1B).

### Pre-slaughtering visit

On the visit day, the temperature in the fattening sector was similar among the farms with the different housing systems, while average values for relative humidity and CO_2_ were higher in the farms with the standard cage and park systems than in those with the dual-purpose cage housing system. The lowest air NH_3_ concentration was recorded in the farms using the enriched cage system (Table 6). Average mortality was numerically higher in the farms with the park system due to the highest value (30.2%) recorded in a single farm on one recording and lower values recorded in the farms using the dual-purpose cage housing system (6.3%) (Table 6).

**Table 6:** Results of the pre-slaughtering visit in farms with different housing systems across three seasons: environmental (means and intervals) and animal-based measures (means) in growing rabbits.

	**Housing system**					**Season**				**RMSE**
	**Standard cage**	**Dual-purpose cage**	**Enriched cages**	**Parks**	***P*-value**	**Autumn**	**Winter**	**Summer**	***P*-value**	
**Environmental data**										
Recordings (no)	9	8	9	7		12	11	10		
Temperature (°C)	20.1 (13.9–28.1)	19.0 (14.5–24.7)	21.0 (17.6–27.4)	20.3 (16.1–26.5)		18.5 (16.1–21.0)	17.2 (13.9–20.7)	25.4 (21.0–28.1)		
Relative humidity (%)	64.4 (43.2–83.6)	57.4 (43.9–74.0)	62.8 (40.4–77.0)	65.2 (49.5–73.5)		65.8 (55.5–83.6)	54.7 (40.4–74.0)	66.8 (58.6–77.0)		
CO_2_ (ppm)	1,221 (480–2,240)	1,063 (520–1,567)	1,048 (640–1,520)	1,297 (540–2,567)		1,334 (600–2,233)	1,452 (740–2,567)	604 (480–680)		
NH_3_ (ppm)	8.6 (0.0–21.0)	8.5 (1.2–21.4)	7.2 (3.4–14.0)	8.5 (1.2–21.7)		9.3 (0.0–21.7)	10.9 (1.2–21.4)	3.8 (1.2–6.2)		
Rabbit mortality (%)	8.9 (4.0–12.7)	6.3 (3.8–9.1)	9.0 (1.4–29.9)	16.5 (7.2–30.2)		8.5 (2.6–20.8)	15.8 (4.5–30.2)	7.6 (1.4–15.9)		
**Animal-based measures**										
Rabbits, no.	900	800	900	700		1,200	1,100	1,000		
Age (days)	71.2	70.1	69.2	71.1		70.3	69.8	71.1		
Live weight (g)	2456^a^	2509^b^	2584^c^	2464^a^	<0.001	2619^a^	2558^b^	2332^c^	<0.001	287.7
Diarrhea (%)*	1.0	0.6	0.0	3.5	1.000	-	0.4	1.9	0.975	
Dermatomycosis (%)*	11.2^a^	32.0^b^	0.3^a^	32.8^b^	<0.001	-	13.0	21.7	0.990	
Injuries (%)*	0.2	0.4	0.5	8.8	1.000	-	0.9	3.1	0.999	

RMSE, root mean square error of the model.

*Prevalence of health concerns is expressed as the average percentage of rabbits affected with respect to the total number assessed each visit at the farm level. Health data for autumn were not recorded.

^a, b, c^ Means with different letters on the same row differ significantly within housing system or season (P-value < 0.05. Bonferroni test).

As for performance, the live weight of growing rabbits decreased from that noted in the farms with the enriched cage to dual-purpose cage system to park and standard cage systems (2,584 vs. 2,509 vs. 2,464 g and 2,456 g; *P* < 0.001). Regarding health issues, a higher prevalence of dermatomycosis was found in farms using the park and dual-purpose cage systems in comparison with those using the standard cage and enriched cage systems (32.8 and 32.0 vs. 11.2% and 0.3% of controlled rabbits), even though these results were linked to a single farm with a very high dermatomycosis occurrence for both the park and dual-purpose cage systems (Table 6). The prevalence of diarrhea in growing rabbits ranged from 0.0 to 3.5%, while injuries were observed in 0.2 to 8.8% of rabbits, without significant differences among housing systems. As for the season, the growing rabbits had lower body weight in summer than in autumn and they were the heaviest in winter (2,332 vs. 2,558 vs. 2,619 kg; *P* < 0.001). No significant difference was observed concerning health issues among the seasons.

The analysis of risk factors for the performance of growing rabbits confirmed the above-described significant effects in reproducing does about the housing system and season, besides genotype (Table 7; Supplementary Table 3). The season was a risk factor for dermatomycosis as well.

**Table 7:** Risk factors (*P-values*) for animal-based measures in growing rabbits at the pre-slaughtering visit in farms with different housing systems across three seasons.

**Variation factors**	**Housing system**	**Season**	**Animal genotype**	**Reproductive rhythm**	**Feeding system**
**Levels**	**Standard/ dual-purpose/ enriched/park**	**Autumn/ winter/summer**	**Grimaud/ Hyla/Martini**	**11 d/ 18 d after kindling**	***Ad libitum*** **/ restricted**
Live weight	<0.001	<0.001	<0.001	0.132	0.018
Diarrhea	0.427	0.015	1.000	0.996	0.992
Dermatomycosis	0.007	<0.001	0.757	0.975	0.006
Injuries	0.035	0.009	0.868	0.978	0.977

Finally, hair cortisol was higher in the growing rabbits housed in the farms with the parks than in those from other housing systems (1.89 vs. 0.93, 0.96, 1.22 ng/g; *P* < 0.001; Figure 1C) and was higher in summer than in autumn (1.55 vs. 0.94 ng/g; *P* < 0.001) (Figure 1D). A significant interaction between housing system × season was observed (*P* < 0.001), i.e., the hair cortisol during summer was higher in rabbits from parks than in those from the other housing systems (2.55 vs. 1.09, 1.02 and 1.55 ng/g; *P* < 0.05), while no significant differences among housing systems were observed in autumn (0.77, 0.89, 0.88, 1.23 ng/g) (Figure 1E).

## Discussion

The present study aimed to provide new information about the on-farm welfare and health of rabbits. Being under field conditions, the sample size per housing system was low due to the availability of farmers and the low number of commercial farms using alternative systems such as enriched cages and park systems. Therefore, not all production factors were fully balanced among the different housing systems. Due to these limits, we first ran a comparison of farms according to the housing systems, considering the farm with its specific combination of production factors as a random effect; then, we used the risk analyses to elucidate the possible main effects of all production factors. Thus, finally, the tested protocol provided only a preliminary evaluation of rabbit welfare and health in farms using the standard and alternative housing systems, whereas recent on-farm assessments focused on those farms using only standard barren cages (15, 16). Moreover, this pilot study highlighted the troubles of accounting for on-farm rabbit welfare and health exclusively to the housing system.

In fact, being recognized and accepting the complexity of the production systems for rabbits (11), the health and welfare of reproducing does and growing rabbits are affected by several factors. Thus, the risk analyses we performed were intended to highlight the role of these factors. The corresponding results are hereby discussed before the comparison of the housing systems.

External factors (such as season), animal-related issues (such as genotype and parity order in does), and management- and structure-based factors (reproductive rhythm, presence of footrest in reproducing cages, and feeding system for growing rabbits) played a significant role.

As for the season, performance results in does, kits, and growing rabbits were lower in summer than in autumn and winter. Indeed, rabbits are very sensitive to high ambient temperatures, since they have few functional sweat glands limiting their ability to eliminate excess body heat (20). Exposure of growing and adult rabbits to severe heat stress adversely affects their growth and reproductive performances as they reduce feed intake to diminish body heat production (21, 22). The highest hair cortisol levels measured in growing rabbits housed in parks during summer suggest that parks can be more stressful for growing rabbits submitted to heat stress, while in autumn, positive effects due to higher available total surface of parks, higher social interaction, and the presence of a plastic-mesh floor prevail. As for doe health, a higher prevalence of diarrhea was observed in autumn than in the other seasons, which could be due to the susceptibility of rabbits to the sudden temperature and air quality changes that are frequent in this season. Interestingly, the same was not observed with regard to respiratory signs. These changes are the main environmental risk factors for diarrhea as identified also by the experts invited to the EFSA technical hearing meeting (11). Additionally, both in reproducing does and growing rabbits, dermatomycosis prevalence was much higher in summer than in autumn and winter. Indeed, according to EFSA (23), dermatomycosis is directly related to environmental factors such as high temperature and humidity, in addition to other factors like low hygienic condition, poor management, and skin lesions (24).

As for animal-related factors, animals belonging to genetic lines selected for growth rate are heavier, have greater feed intake, and better feed conversion than those from lines selected for litter size (25–28). In the present study, Hyla females were heavier than Grimaud and Martini females, the latter being present only in one farm, whereas Grimaud litters were larger and Grimaud kits and growing rabbits were heavier than Hyla and Martini ones (Supplementary Table 2), which is consistent with the observations in the study of Martínez-Bas et al. (29). Differently, Zita et al. (30) reported a higher weaning and slaughtering weight in Hyla compared to Grimaud rabbits. Under our conditions, genotype was not associated with any major risk for health issues. In contrast, previous authors (31, 32) found a relationship between genotype and prevalence of pododermatitis in commercial farms with conventional housing systems, with those with the heavier strain at a higher risk of pododermatitis. A relationship with genotype was also previously reported for the prevalence of clinical mastitis in commercial farms (18).

As already found in the literature (33), our results showed that performance changed with parity order, with multiparous does being heavier and having larger litters compared to primiparous ones (Supplementary Table 2). Also, in our trial, kit weight and weight gain increased with the parity order of reproducing does due to their higher feed intake and, accordingly, higher milk production. Moreover, based on the literature (26), the longer the reproductive career, the lower the BCS of the doe. Under our conditions, parity order was also found to be a risk for pododermatitis occurrence but not for mastitis, which is consistent with the results of Rosell et al. (32).

With regards to management factors, as for the reproductive rhythm, there are several studies comparing doe and litter performance and doe body energy balance using intensive (insemination post-partum or 11–12 days after kindling) or extensive rhythms (insemination after litter weaning), while rhythms based on insemination 17–19 days after kindling have become popular in the field without evidence of data in the literature (33). Under our conditions, in the tested farms using the 11-day rhythm, does had higher BCS and larger litters at weaning than in farms inseminating does 18 days after kindling (Supplementary Table 2), which is quite surprising and would deserve further investigation under experimentally controlled conditions. It could be hypothesized that the ongoing pregnancy in females submitted to the 11-day rhythm accounts for their higher BCS to ensure future offspring compared to females submitted to the 18-days rhythm. Based on the literature (18), the reproductive rhythm is a risk factor also for the occurrence of pododermatitis, mastitis, and dermatomycosis in reproducing does. In fact, Rosell and de la Fuente (18) reported that diseases (mastitis) or worse BCS are predisposing risk factors for infertility, whereas the reproductive rhythm can affect overall farm productive results. Thus, fertility might be included as a further indicator in protocols for on-farm welfare and health assessment. The prevalence of clinical mastitis is also affected by the lactation stage; as in commercial farms, clinical mastitis was found to be more frequent in the fifth week of lactation compared to the first one (18).

Our results showed that, among factors linked to housing, the absence of footrest mats was a risk factor for the occurrence of pododermatitis and dermatomycoses in reproducing does, which confirms the usefulness of such a tool (12, 32, 34).

### Welfare and health of reproducing does and litters in different housing systems

We used the criteria established in the Welfare Quality Project (14) (Good Feeding, Good Housing, Good Health, and Appropriate Behavior) as a reference for identifying indicators for on-farm measurements. Behavioral concerns and constraints were implicitly assessed by resource- and management-based indicators since there is no doubt about how cage type, group size per cage, and the presence of enrichments can affect movement restriction, resting problems, and expression of social and gnawing behaviors. Moreover, negative behaviors, such as aggression, were assessed based on ABMs, i.e., injuries.

Under our conditions, based on cage dimensions, movement restrictions/resting problems were expected in the standard cage and dual-purpose cage housing systems and to a lesser extent in the enriched cage systems and the single modules of parks for reproducing does. As regards social behaviors, reproducing does were kept with their litters from kindling until weaning, experiencing individual housing for about 7 to 10 days, depending on the reproductive rhythms (kindling to kindling interval: 42 or 49 days; i.e., 17 and 20% of the time covering a reproductive cycle, respectively). Moreover, in the tested farms, does were never kept with other adult mates. Finally, no gnawing object was found in cages or parks of the visited farms for which rabbits were not able to play this species-specific behavior on any farm.

According to EFSA (11), despite the above-stated differences in available areas for movement, the main welfare consequence for reproducing does in both standard cages and dual-purpose cages, enriched cages, and parks is the restriction of movement, defined as the possibility of performing three consecutive hops. However, again according to EFSA (11), knowledge is missing about the space requirement to acceptably meet the behavioral and physiological needs of rabbits under farming conditions. Moreover, more space and locomotion possibilities can affect doe performance on-farm (12, 35): some authors (36) observed higher body weight and weight gain in does housed in conventional cages than those kept in larger cages, while others reported few differences (35). In the case of reproducing does, an impairment in performance is especially expected when comparing conventional individual housing with collective housing systems, which has been related to aggression and stressful interactions among does rather than to space availability itself (37, 38).

Under our conditions, the lowest live weight of the reproducing does in farms with the standard cage system and their lowest body condition score compared to those in dual-purpose cages and, especially, in enriched cages and parks cannot be associated with differences in the genotype distribution or in the reproductive rhythm used or in the distribution of primiparous and multiparous does in the farms using the different housing systems (Supplementary Tables 1, 2). In fact, as for the genotype, as presented above, the heavier Hyla females and the lighter Grimaud and/or Martini females were present in all housing systems. As for the reproductive rhythm, the highest BCS and the largest litters at weaning have been associated with the 11-day rhythm compared to the 18-day rhythm where the former was prevalent in farms with standard cages (two out of three farms) and enriched cages (three out of three farms) compared to farms with dual-purpose cages (one out of three farms) and parks (one out of three farms). Finally, as for the parity order, the percentage of primiparous does used in the evaluation was similar in all farms (10–15% of the total). Nevertheless, the parity order of the doe can play a major role in her status. It would be recommendable to include in the evaluation only does with more than three kindlings, which would represent the majority of the does on the farm and would be in a more stable condition compared to does at the start of their reproducing career. Weaning weight can also affect the adaptability and survival of rabbits after weaning in the growth period until slaughtering (39, 40). In the literature, some studies observed worse litter performance in larger cages (41), while others observed heavier kits in larger cages with an elevated platform compared to smaller cages without platforms (42) which was ascribed to a higher disturbance to the sleeping of kits due to doe visits in the nest boxes (more than two nursing events/day) in smaller cages. When focusing on health-related welfare consequences in reproducing does, EFSA (11) ranked heat stress as one of the top five welfare consequences in conventional standard and dual-purpose cages and enriched cages and skin lesions in parks. Indeed, we did not detect any difference in the occurrence of health concerns both for does and litters among the farms with different housing systems. Moreover, a previous study found a higher occurrence of mastitis and diarrhea in larger cages, which was due to the higher soiling of the floor because of an unsuitable footrest mat (36).

As for kits, EFSA (11) ranked hunger as the main welfare concern in conventional cages and parks; neonatal disorders are ranked only for parks, while heat stress, neonatal disorders, and respiratory disorders have been alternatively listed in the three housing systems tested in the present study. However, in the present study, no signs of hunger and neonatal or respiratory disorders were detected, whereas heat stress was likely to occur only during summer in all housing systems, as measured by the low kit weaning weight during this season. In fact, the indoor maximum temperatures we measured during the visits ranged from 24.7 to 28.6°C, which is somewhat higher than the optimal ranges for reproducing does and litter, i.e., 15–20°C, 60–70% humidity (43), while severe heat stress is known to occur above 30°C (43, 44). Under our conditions, air CO_2_ and NH_3_ did not exceed the recommended thresholds for farms, i.e., 5,000 ppm and 25 ppm, respectively (44, 45), with higher values recorded in winter than in summer. These results are consistent with the observations of Calvet et al. (46) and with the Italian climate conditions for which farm air changes are lower during winter to maintain temperature, which produces a worse air quality, even if always within acceptable ranges (47).

### Welfare and health of growing rabbits in different housing systems

As for behavioral constraints in growing rabbits, according to EFSA (11), inability to express gnawing behavior and resting problems are the main welfare consequences in all the housing systems we compared, while the restriction of movement is ranked in cages but not in parks.

In fact, no gnawing objects were found in cages or parks in which rabbits were prevented from gnawing in all tested farms. Moreover, based on cage size and stocking density (16–17 rabbits/m^2^ in standard bicellular cages, dual-purpose cages, and enriched cages; 12 rabbits/m^2^ in parks), restriction of movement and resting problems were likely to occur in cage systems compared to parks.

As for the differences found in the final live weight of growing rabbits, taking into account also differences in slaughter age, the best performance was found in the rabbits kept in farms using the dual-purpose cage and enriched cage housing systems compared to those using standard bicellular cages and parks. These results cannot be attributed to differences in the genotype (since the heavier Grimaud and the lighter Hyla and/or Martini growing rabbits had the same distribution in all housing systems) (Supplementary Tables 1, 3). Also, the nonhomogeneous distribution of the feeding system cannot alone explain the differences in the live weight of growing rabbits in the different housing systems. In fact, the heaviest animals were feed-restricted (two out of three farms with dual-purpose cages) and fed *ad libitum* (three out of three farms with enriched cages) as it was for the lightest animals which were both restricted (two out of three farms with standard cages) and *ad libitum* fed (three out of three farms with parks). Even if the best growth performance is not necessarily associated with the best welfare conditions, it is likely that movement restrictions in bicellular cages were too high to favor non-active behaviors and reduce feeding. This hypothesis is supported by the high stocking density (as kg live weight at slaughtering) recorded on farms using standard bicellular cages, i.e., on average 46.0 kg/m^2^ (from 33 to 56 kg/m^2^), which can support the “prolonged hunger” ranked by EFSA (11) within the top five welfare consequences for growing rabbits in conventional cages. Moreover, interactions within large groups of animals and high movement possibilities could have reduced feed intake and growth in parks. Indeed, even rabbits kept in small groups have been observed to spend more time moving and less time feeding than rabbits in bicellular cages, which can affect performance (48). More space and locomotion possibilities, i.e., greater physical activity, can also have a negative impact on performance (49–51). However, recent studies showed higher daily weight gain and final live weight in rabbits reared in large groups (58 rabbits) compared with rabbits reared in small groups (12 rabbits) in the first growth period (until 60 days) (52). Thus, based also on the low stocking density measured in farms using park housing systems at slaughtering (on average 30.1 kg/m^2^; range: 29–32 kg/m^2^), a high degree of social interactions due to the group size (32–40 rabbits per group) likely decreased feed intake and growth in parks of the visited farms rather than behavioral restrictions.

As for health concerns, according to EFSA (11), skin and gastrointestinal disorders are among the top five welfare consequences in rabbits farmed in enriched cages and parks, while in the present study, only a higher prevalence of dermatomycosis was observed in farms using the dual-purpose cage and park housing systems and no effect of the housing system was reported for diarrhea on a small sample size of farms which require confirmation on a larger scale.

At the pre-slaughtering visit, injuries due to aggressive behavior were recorded; the occurrence of injured rabbits was numerically higher in farms using the park system (8.8 vs. <1%) but the difference was not confirmed at a statistical level. It is widely reported in the literature that aggressions are positively correlated with increased group size, stocking density, and slaughtering age (33, 49, 53, 54). Accordingly, stress is expected to increase with the group size as higher corticosterone levels in hair and feces have been measured in rabbits kept in collective pens compared to rabbits in bicellular cages when age increased (from 63 to 70 days) in previous studies (48). These results are consistent with the increased hair cortisol we measured in growing rabbits housed in parks during summer when temperature/humidity was likely more challenging compared to autumn, as discussed above.

## Conclusion

Despite preliminary testing, because of the low sample size per farm type and the field conditions, the tested on-farm protocol did not highlight major differences in welfare and health of reproducing does and their kits or growing rabbits kept in different housing systems. Few differences for health concerns were recorded among housing systems, whereas neither lesion in growing rabbits due to aggression significantly changed in collective systems with a high group size, such as parks. Importantly, the study outlined the role of several production factors changing from one farm to another, stressing the troubles of accounting on-farm rabbit welfare and health exclusively to the housing system. In perspectives, interactions between these factors and the housing systems should be highlighted to improve the whole production system; on-farm protocols should be refined based on the sensitivity of AMBs to production factors other than the housing system; and ABMs based on feelings should be identified and validated to provide additional tools for evaluating on-farm welfare of rabbits.

## Data availability statement

The original contributions presented in the study are included in the article/Supplementary material, further inquiries can be directed to the corresponding author.

## Ethics statement

This study was conducted in compliance with Council Directive 98/58/EC concerning the protection of animals kept for farming purposes, enacted in Italy through the Legislative Decree No. 146/2001 and approved by the Ethics Committee of the Istituto Zooprofilattico Sperimentale delle Venezie (CE_IZSVE 6/2022 of 4 July 2022).

## Author contributions

AT, GDM, and GX contributed conception and design of the study. CZ, DP, GC, FD and FP collected data on-farms. AL, DB and MB performed lab analyses. CZ performed the statistical analysis. AT and FM wrote the first draft of the manuscript. All authors contributed to manuscript revision, read, and approved the submitted version.

## Funding

This research was funded by the Italian Ministry of Health (Project IZSVe RC16/17; id: B23C17000190001).

## Conflict of interest

The authors declare that the research was conducted in the absence of any commercial or financial relationships that could be construed as a potential conflict of interest.

## Publisher's note

All claims expressed in this article are solely those of the authors and do not necessarily represent those of their affiliated organizations, or those of the publisher, the editors and the reviewers. Any product that may be evaluated in this article, or claim that may be made by its manufacturer, is not guaranteed or endorsed by the publisher.
