# Supplementary material for: A pilot study about on-farm assessment of health and welfare in rabbits kept in different housing systems
Source: Front Vet Sci. 2022 Aug 11;9:936643. doi: 10.3389/fvets.2022.936643 (PMC9403509; doi:10.3389/fvets.2022.936643)
Supplement: Supplementary file 1 [file Data_Sheet_1.docx]

**Table S1.** Housing systems and cage size in the farms subjected to the on-farm welfare evaluation in reproducing does with their litters and in growing rabbits

| Farm ID | Cage | Genotype | Reproductive rhythm | Weaning age | Slaughtering age | Footrest mat | Age of cages | Feeding program for growing rabbits | Ventilation  Cooling system |
| --- | --- | --- | --- | --- | --- | --- | --- | --- | --- |
| A | Standard | Hyla | 11 | 32 | 84 | No | 1983 | Restriction | Longitudinal  No cooling |
| B | Standard | Grimaud | 18 | 35 | 80 | No | 2006 | Restriction | Longitudinal Cooling |
| C | Standard | Hyla | 11 | 37 | 75 | No | 1998 | Ad libitum | Longitudinal |
| D | Dual purpose | Hyla | 18 | 35 | 78-80 | No | 2010 | Restriction | Longitudinal Cooling |
| E | Dual purpose | Grimaud | 18 | 35 | 78 | Yes | 2015 | Ad libitum | Transversal Cooling |
| F | Dual purpose | Hyla | 11 | 37 | 74-76 | No | 2009 | Restriction | Longitudinal Cooling |
| G | Enriched | Hyla | 11 | 35 | 72 | No | 2017 | Ad libitum | Longitudinal Cooling |
| H | Enriched | Martini | 11 | 35 | 72 | Yes | 2008 | Ad libitum | Transversal Cooling |
| I | Enriched | Hyla | 11 | 35 | 74 | Yes | 2017 | Ad libitum | Transversal Cooling |
| L | Park | Hyla | 11 | 37 | 73-75 | No | 2008 | Ad libitum | Longitudinal |
| M | Park | Hyla | 18 | 35 | 70 | Yes | 2015 | Ad libitum | Longitudinal Cooling |
| N | Park | Grimaud | 18 | 38 | 78-79 | Yes | 2017 | Ad libitum | Longitudinal Cooling |

**Table S2.** Mean values for animal-based measures in reproducing does and kits at the pre-weaning visit in farms with different housing systems across three seasons based on animal genotype, reproductive rhythm, parity order and footrest presence

|  | Animal genotype | | |  | Reproductive rhythm | |  | Parity order | |  | Footrest presence | |
| --- | --- | --- | --- | --- | --- | --- | --- | --- | --- | --- | --- | --- |
|  | Grimaud | Hyla | Martini |  | 11 d after kindling | 18 d after kindling |  | Primiparous | Multiparous |  | Yes | No |
| Doe |  |  |  |  |  |  |  |  |  |  |  |  |
| Live weight (g) | 4673 | 4870 | 4461 |  | 4775 | 4745 |  | 4638 | 4956 |  | 4943 | 4631 |
| Body condition score | 1.93 | 1.99 | 1.96 |  | 1.98 | 1.95 |  | 1.93 | 1.98 |  | 2.05 | 1.91 |
| Diarrhea (%)* | 3.66 | 6.87 | 6.67 |  | 6.91 | 4.58 |  | 5.99 | 5.96 |  | 5.44 | 6.34 |
| Pododermatitis (%)* | 1.83 | 4.71 | 0.53 |  | 1.98 | 5.14 |  | 1.51 | 7.30 |  | 0.33 | 5.19 |
| Mastitis (%)* | 1.50 | 3.49 | 2.40 |  | 2.15 | 3.72 |  | 1.65 | 5.41 |  | 0.67 | 4.19 |
| Dermatomycosis (%)* | 2.17 | 1.65 | 8.26 |  | 3.33 | 2.22 |  | 2.92 | 2.82 |  | 1.67 | 3.70 |
| Litter |  |  |  |  |  |  |  |  |  |  |  |  |
| Litter size (no.) | 9.16 | 8.21 | 8.28 |  | 8.35 | 8.66 |  | 8.51 | 8.57 |  | 8.99 | 8.11 |
| Average kit weight (g) | 660 | 614 | 549 |  | 592 | 652 |  | 612 | 625 |  | 613 | 618 |
| Diarrhea (%)* | 1.83 | 0.96 | 0.00 |  | 0.67 | 1.58 |  | 1.27 | 0.48 |  | 1.78 | 0.54 |
| Dermatomycosis (%)* | 1.67 | 2.19 | 0.27 |  | 0.51 | 3.56 |  | 1.01 | 3.41 |  | 2.88 | 0.00 |

*Percentage of animals affected with respect to the total assessed on each visit per farm

**Table S3.** Mean values for animal-based measures in growing rabbits at the pre-slaughtering visit in farms with different housing systems across three seasons based on animal genotype, reproductive rhythm, and feeding system

|  | Animal genotype | | |  | Reproductive rhythm | |  | Feeding system | |
| --- | --- | --- | --- | --- | --- | --- | --- | --- | --- |
|  | Grimaud | Hyla | Martini |  | 11 d after kindling | 18 d after kindling |  | Ad libitum | Restricted |
| Live weight (g) | 2567 | 2448 | 2375 |  | 2459 | 2468 |  | 2512 | 2418 |
| Diarrhea (%)* | 0.60 | 1.82 | 0.00 |  | 1.43 | 0.43 |  | 1.77 | 0.00 |
| Dermatomycosis (%)* | 0.00 | 32.6 | 0.20 |  | 14.3 | 22.9 |  | 15.3 | 20.1 |
| Injuries (%)* | 0.20 | 3.45 | 0.40 |  | 2.79 | 0.29 |  | 3.00 | 0.25 |

*Percentage of animals affected with respect to the total assessed on each visit per farm


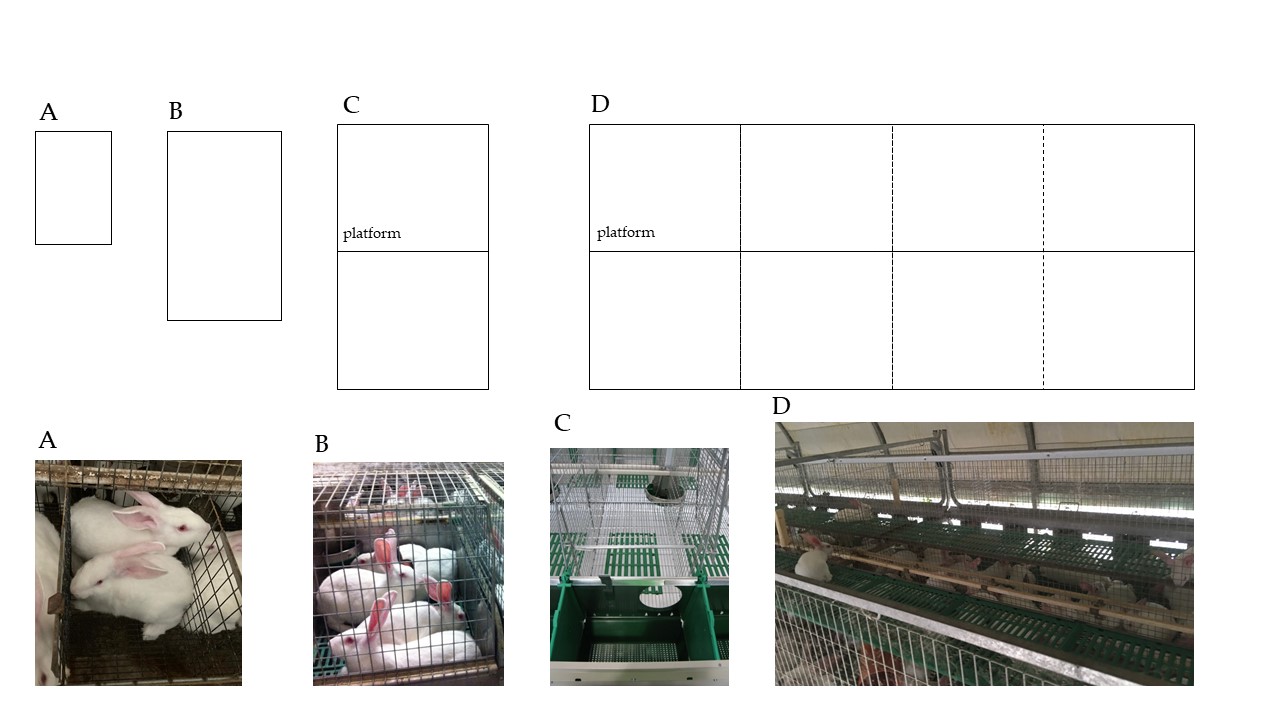


Figure S1: Schemes and pictures referred to A) Bicellular cage, B) Dual-purpose cage, C) WRSA, D) Park.
